# DNA double strand break repair: a model of specificity and complexity in SUMO signalling

**DOI:** 10.1042/EBC20253043

**Published:** 2025-12-12

**Authors:** Jai S Bhachoo, Alexander J Garvin

**Affiliations:** 1 SUMO Biology Lab, School of Molecular and Cellular Biology and Astbury Centre for Structural Molecular Biology, Faculty of Biological Sciences, University of Leeds, Leeds, U.K

**Keywords:** chromatin, DNA synthesis and repair, SUMOylation, ubiquitin ligases, ubiquitin proteasome system, ubiquitin signalling

## Abstract

Among the ubiquitin-like superfamily, small ubiquitin-like modifiers (SUMOs) are the most well-understood. However, in comparison with the prototypical small modifier ubiquitin, our understanding of the SUMO system lags. SUMOylation is often characterised as ‘simple’ in comparison with ubiquitination, with fewer SUMO-specific writers, readers and erasers compared with the ubiquitin machinery. A key divergence between ubiquitin and SUMO is that the SUMOylation system utilises a group of related SUMOs (SUMO1– 5), each possessing distinct functions. SUMO paralogs share conjugation, recognition and deconjugation machinery, yet signalling can employ each to perform specific cellular functions. This illustrates a complex layer of molecular discrimination that is far from simple. The repair of DNA double-stranded breaks (DSBs) – highly toxic DNA lesions generated from both endogenous and external sources – serves as a fascinating exemplar of specificity in SUMO signalling. This review focuses on how signalling specificity is achieved during SUMO-DSB repair. Examples of how different branches of SUMO signalling can direct discrete DSB-repair outcomes through modulation of key repair factors, including the RAP80-BRCA1-A complex, RNF168 and CtIP, are described in further detail.

## The SUMO family

### SUMO1, the ancestral SUMO

**Figure 1:**
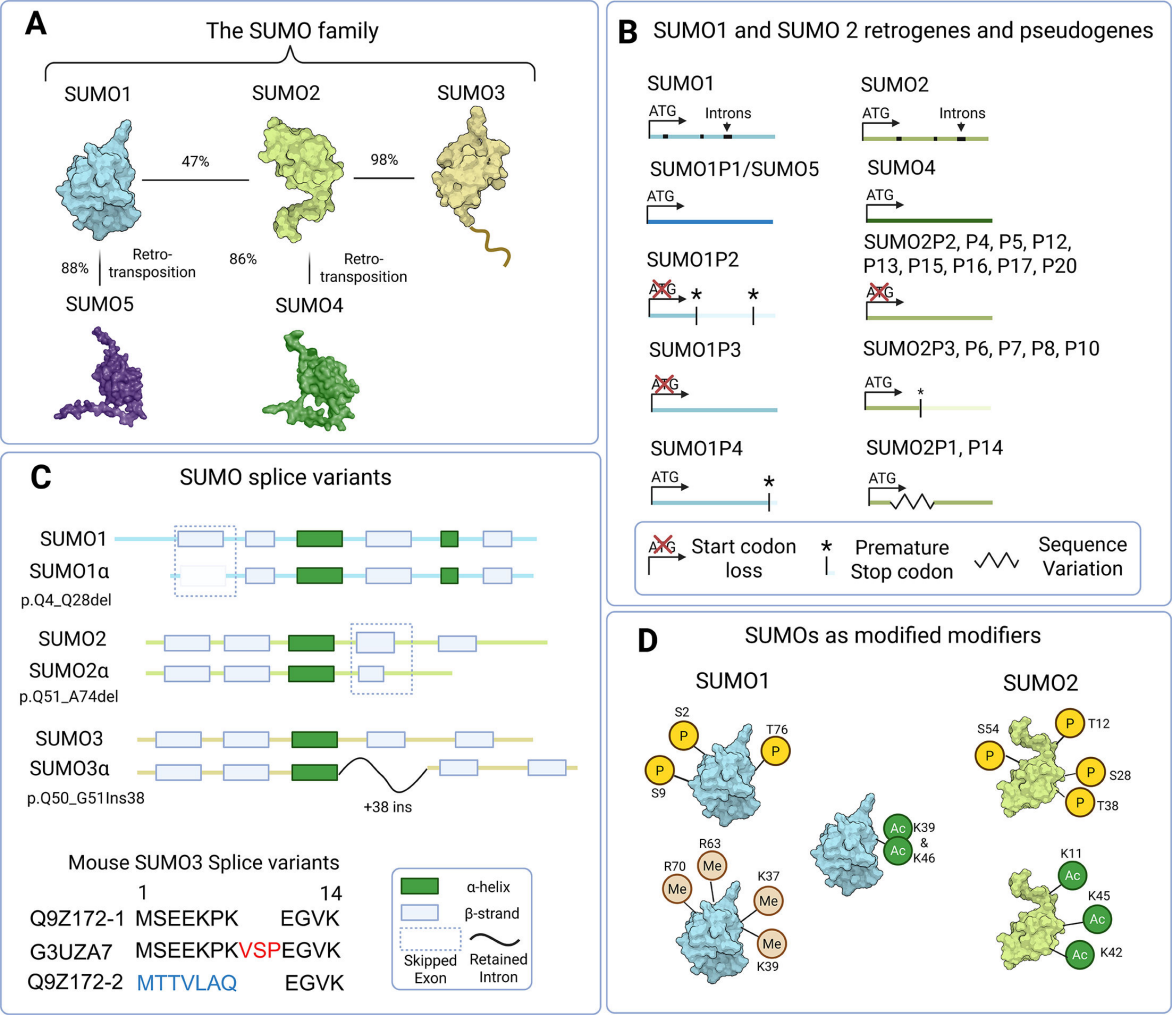
The larger SUMO family. A: The five SUMO family members. Amino acid similarity percentages between SUMO2 and SUMO3=96.8%, and between SUMO1 and SUMO2=47.3%, SUMO4 and SUMO5 are products of retrotransposition from SUMO2 and SUMO1, respectively. NMR solution structures are shown for SUMO1 (PDB file: 2N1V), SUMO2 (PDB file: 2N1W), and SUMO3 (PDB file: 1U4A). AlphaFold predicted structures for full-length SUMO4 (AF-Q6EEV6-F1-v4) and SUMO5 (AF-G2XKQ0-F1-v4) are shown and visualised using Pymol. B: SUMO1 and SUMO2 retrogene and pseudogene schematics (not to scale). The approximate locations of introns are shown for SUMO1 and SUMO2. There are no annotated SUMO3 pseudogenes in the human genome. C: SUMO splice (α-variants) variants for human SUMO 1, 2, 3 and mouse SUMO3. Q9Z172-1 is the canonical mouse SUMO3 transcript. G3UZA7 has a VSP insertion, whereas Q9Z172-2 has a divergent N-terminus. D: SUMOs as modified modifiers. SUMO can be modified by other PTMs such as phosphorylation, acetylation and methylation. The location of PTMs is approximate, and not all sites are illustrated.

Small ubiquitin-like modifier 1 (SUMO1) shares only ~50% homology with other SUMO paralogs (Figure 1a). Unlike SUMO2/3, SUMO1 can compensate for the loss of SMT3 in *S. cerevisiae*, confirming functionality akin to SUMOs in single-cell eukaryotes. SUMO1 accumulates at the nuclear periphery, where a substantial fraction stably conjugates to RanGAP1. SUMO1 conjugates are therefore less dynamic in stress-responsive conjugation compared with SUMO2/3 [1]. During double-stranded break (DSB) induction, bulk changes in SUMO1ylation are less evident than SUMO2/3ylation, but SUMO1 does modify multiple DSB repair factors and has essential and distinct signalling roles versus SUMO2/3 during DSB repair [2].

### SUMO2/3, the (almost) identical twins

SUMO2/3 are highly related paralogs; immature proSUMO3 differs from proSUMO2 by a longer C-terminal tail, which is cleaved before conjugation. Mature SUMO2/3 proteins differ by only two amino acids, so they cannot be distinguished by antibodies and are referred to as SUMO2/3 [3,4]. In most cell types, SUMO2 mRNA and protein are more abundant than those of SUMO3 [5]. In mice, SUMO2^KO^ is embryonic lethal, while SUMO3^KO^ are viable with minimal phenotypic abnormalities [6]. Some molecular discrimination between SUMO2 and SUMO3 may exist, as specific interactors have been identified, suggesting some ability to ‘read’ the small N-terminal variations between SUMO2 and SUMO3 [7]. Differences in deSUMOylation of SUMO2 and SUMO3 N-terminal peptide models have also been detected *in vitro* [8]. While highly similar, it is therefore possible that SUMO2 and SUMO3 perform subtly different cellular functions.

### SUMO4 the pseudoSUMO

*SUMO4* is an ape-specific retrogene (Figure 1b) [9]. Like other retrogenes, SUMO4 mRNA and protein are expressed at significantly lower levels compared with the highly abundant SUMO1–3. A defining biochemical feature of SUMO4 is the presence of Pro-90 (equivalent to Gln-90 in SUMO2), which prevents SENPs from cleaving the last two residues of SUMO4, leaving it trapped in its immature, unconjugatable state [10]. Stress-responsive, immune-related and NFκB signalling roles have been attributed to SUMO4; however, issues with the usage of SUMO2/3/4 cross-reactive antibodies and artificially matured SUMO4 cDNA complicate the interpretation of its function [2,3,11]. Differentiating SUMO4 tryptic peptides from SUMO2/3 in mass spectrometry is also nearly impossible [12]. We recently described an atypical, conjugation-independent role for endogenous SUMO4, which stimulates the SUMO protease SENP1 and regulates global SUMO1-3ylation dynamics essential for efficient double-strand break (DSB) repair. Retaining the SUMO binding features of its SUMO2 ancestor but lacking conjugation ability, we define SUMO4 as a ‘pseudoSUMO’ – similar to pseudokinases that maintain regulatory features while losing typical enzymatic functions [2].

### SUMO5 and SUMO pseudogenes

Humans have four SUMO1 pseudogenes; three have either lost their start codon or have premature stop codons (Figure 1b). SUMO5/SUMO1P1 is the only intact pseudogene. Like SUMO4, it is an evolutionarily modern retrogene with low-level mRNA and tissue-specific expression [5,13]. SUMO5 has been proposed to regulate PML nuclear body dynamics. However, a lack of detection reagents to specifically detect SUMO5 in human cells makes a demonstration of endogenous function uncertain. More research is needed to establish if SUMO5 is a faithful protein-coding member of the SUMO family. Humans have a further 17 *SUMO2*-related pseudogenes, all of which have lost start codons, gained premature stop codons, have large deletions or exhibit highly divergent amino acid sequences. Thus, it is unlikely that any retain SUMO protein-related functions, though further analysis is necessary to confirm this [14].

### The alphas, SUMO1–3 splice variants

The splicing of SUMO1–3 (alpha variants) further expands the SUMO family (Figure 1c). SUMO1α and SUMO2α are primarily cytoplasmic and less capable of forming conjugates, whereas SUMO3α can form conjugates and has a localisation that is more similar to the canonical SUMO3 [15]. Differences in expression of these splice variants during stress responses suggest that splicing has a role in further diversifying SUMO signalling [15]. In mice, SUMO3 is expressed as three conjugatable isoforms; their functional significance remains to be determined [16].

### SUMOs as modified modifiers

SUMOs also undergo PTMs, including SUMOylation, ubiquitination, phosphorylation, methylation and acetylation (Figure 1d) [17]. Phosphorylation of SUMO1^Ser2^ has been detected in multiple phosphoproteomic screens, but its function remains unclear [18]. SUMO1^Thr76^ phosphorylation by Akt increases the stability of unconjugated SUMO1 [19]. SUMO1^Ser9^ phosphorylation aids the unstructured N-terminus to act as an autoinhibition domain in folding over the SUMO-interacting motif (SIM) binding groove, blocking SUMO1’s ability to interact with SIM motifs [20,21].

### SUMO conjugation

**Figure 2:**
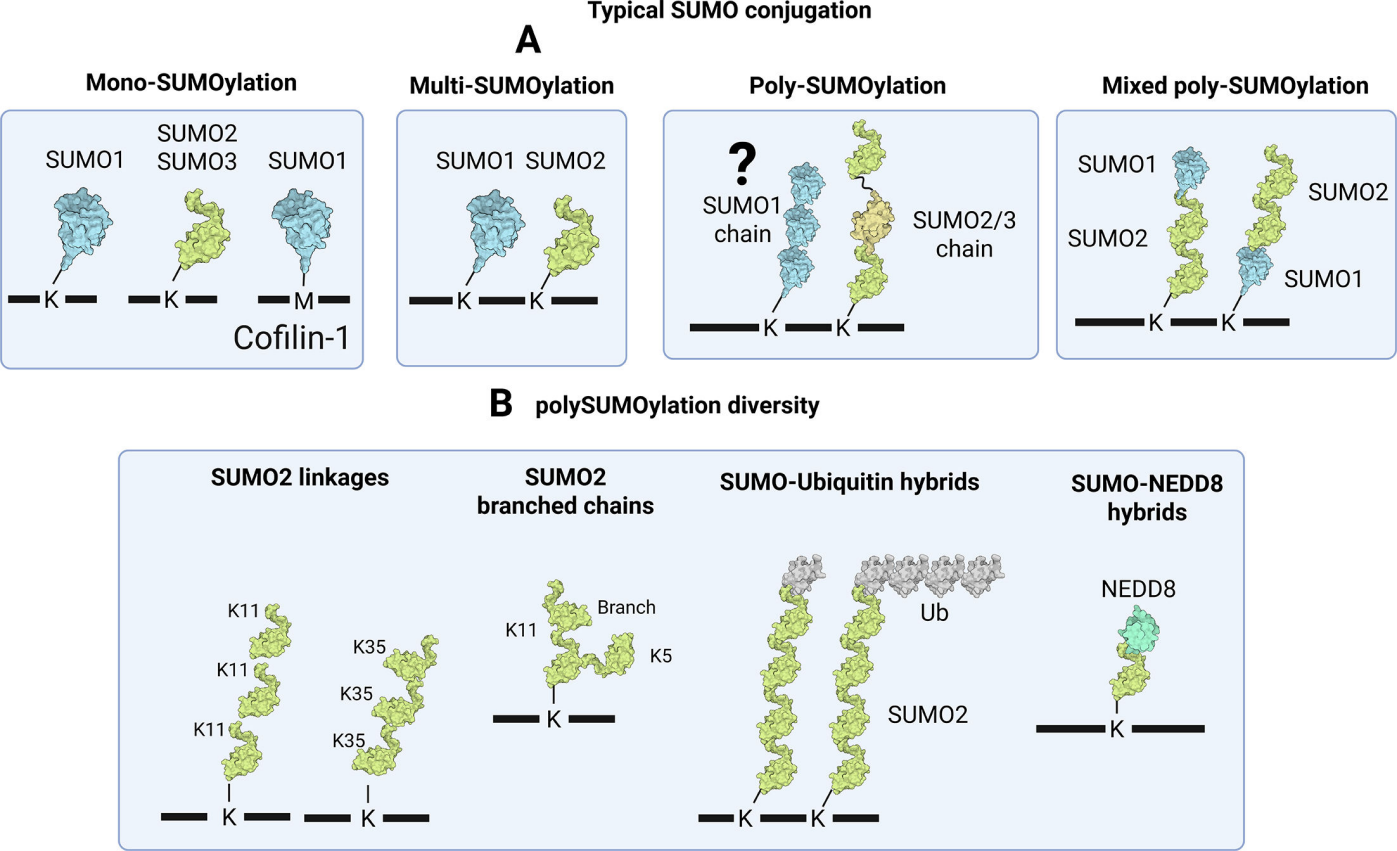
SUMO conjugation. A: Substrates can be modified by the three confirmed conjugatable SUMOs. This can be in the form of mono-SUMOylation (singular SUMO modification), multi-SUMOylation (multiple SUMO modifications of the same or mixed SUMO paralog), or poly-SUMOylation (SUMO chains) in the form of mixed/heterogeneous or homogeneous chains. B: SUMOs can form chains on all lysine residues; only two chain types are shown. It is unknown what 3D topology these chains form. Branched chains occur when two SUMOs are linked through different lysines on the same SUMO. Hybrid chains can occur where ubiquitin or NEDD8 are incorporated into SUMO chains or vice versa. The NEDD8 symbol is derived from PDB 1NDD.

SUMOylation employs E1-E2-E3 machinery like ubiquitin but uses a single E2 (Ubc9/UBE2I) and far fewer E3s [22]. SUMOylated lysines often reside in unstructured solvent-exposed regions within consensus sequences ψKxE (ψ=hydrophobic amino acids) [16], enabled by the E2’s facilitating SUMOylation independently of an E3 [23]. Various SUMOylation consensus iterations exist, and adjacent PTMs like phosphorylation, prolyl isomerisation, acetylation and methylation can influence SUMOylation [24–27]. Yet, ~50% of SUMOylated lysines are non-consensus [28], potentially driven by E3 ligase specificity. Non-lysine SUMOylation has been observed for cofilin, occurring at the N-α-NH2 methionine (Figure 2a). This unusual SUMOylation may account for some proteins, even when mutated in all SUMOylatable lysines, or that naturally contain no lysines, such as p14ARF, still becoming SUMOylated [29,30].

SUMOs conjugate to proteins at single sites (mono-SUMOylation), multiple sites (multi-mono-SUMOylation), and themselves (polySUMOylation) (Figure 2). Many proteins can be SUMOylated by both SUMO1 and SUMO2/3 at the same lysine, suggesting a degree of compensatory cross-talk between these two branches of the SUMO family. For example, SUMO1^KO^ mice are viable, indicating that SUMO1 conjugates can be substituted with SUMO2/3. However, proteomics indicates that some substrates exhibit clear paralog conjugation preferences [31]. The E1-SAE2 subunit can contribute to SUMO paralog conjugation preference. Acetylation of Lys^164^ biases SUMO2/3 conjugation over SUMO1 due to the presence of Glu^93^ in the SUMO1 C-terminus (Gln^89/88^ in SUMO2/3). HDAC6-dependent deacetylation during mitosis promotes a SUMO1 conjugation bias essential for mitotic fidelity [32]. The SUMO E3s also impart paralog conjugation specificity as E3s such as ZMIZ2 and ZNF451 show SUMO2/3 bias, while other E3s such as TOPORS, PIAS1, PIAS2 and NSMCE2 show some degree of SUMO1 preference [33,34].

### SUMO polymers

A consensus SUMOylation site surrounding K11 in SUMO2/3’s N-terminus promotes SUMO2/3 chain formation [35]. However, SUMO2/3^K11^ is not the only internal linkage site; all SUMO2/3 lysines can form chains to varying extents. SUMO2/3^K11^ predominates in some cases, while SUMO2/3^K21^ and SUMO2/3^K33^ chains dominate in specific tissues and stress conditions (Figure 2b) [16]. Through site competition, acetylation of SUMO2^K11ac^ can redistribute SUMO2 chain formation to ‘atypical’ non-K11 residues, suggesting a molecular mechanism by which cells can produce other chain types [36]. There is also some indication that different SUMO E3s can promote formation of different linkage types [34]. PolySUMOs can be branched or mixed with SUMO1, ubiquitin or NEDD8 [37–39], further increasing their heterogeneity. SUMO1 lacks a classical SUMOylation consensus site and is proposed to cap SUMO2/3 polymers, limiting their length [40]. However, SUMO1 contains an inverted SUMOylation motif, and large-scale proteomic analysis has identified SUMO2/3ylation of SUMO1 at multiple sites [41].

### SUMO deconjugation

DeSUMOylases include six SENPs (SENP1–3 and SENP5–7), deSUMOylating isopeptidase (DeSI) 1/2 and ubiquitin-specific protease like 1 (USPL1) [42–46]. SENP1 and SENP2 have endopeptidase activity, wherein the SUMO1–3 precursor tails are cleaved to expose terminal -GG residues [47]. SENP1 and SENP2 deSUMOylates SUMO1–3 and broad conjugate types [48–50], SENP3, SENP5 and USPL1 show preference for SUMO2/3 conjugates, while SENP6 and SENP7 are SUMO2/3 chain editors with limited reported activity against SUMO mono-modifications [51–54]. *In vitro,* the SENP3 catalytic domain has little activity against model substrates; rather, it requires allosteric stimulation by a short linear motif from its binding partner PELP1 [55]. The specificities of DeSI1/2 proteases are less characterised [43,56]. DeSUMOylase catalytic domains, therefore, have some degree of intrinsic specificity in regulating the type and paralog specificity of their SUMO conjugate substrates. Further nuance into SENP specificity can be appreciated through the use of active site probes in the context of full-length SENPs from cell lysates or isolated SENP catalytic domains. Labelling of SENP3–7 by SUMO1 probes has been detected, suggesting that SENP3–7 may have broader paralog specificity than is currently appreciated. DiSUMO2 probes label all full-length SENPs except SENP5 [2,48,57–60]. This is in line with findings that most SENPs possess polySUMO deconjugation activity, with SENP1 activity exceeding that of SENP6 and SENP7 [48]. As full-length recombinant SENPs have not been purified, these studies suggest that sequences outside the catalytic domain may contribute to SENP-SUMO paralog specificity. A screen of monoSUMOylated peptides also confirms that, *in vitro,* the catalytic domains of SENP6 and SENP7 have some degree of monoSUMO3 deconjugation activity, at least *in vitro* [8].

**Figure 3:**
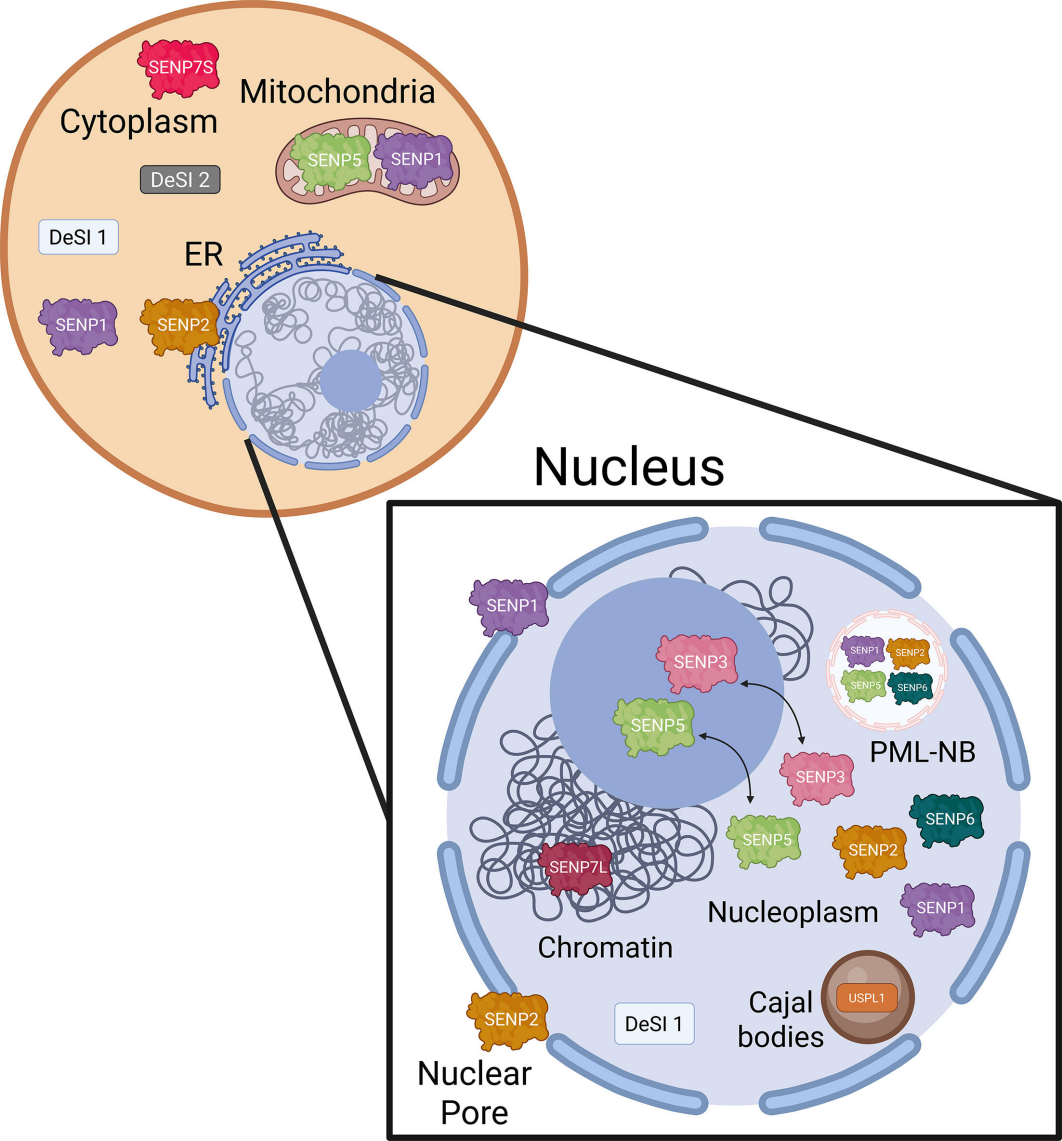
DeSUMOylases cellular localisation. DeSUMOylases localise to various compartments of the cell, with the majority highly concentrated in the nucleus and its associated compartments. SENPs are also found outside of the nucleus. SENP1 and SENP5 have been observed to localise to mitochondria [61]. SENP1 and SENP2 localise to nuclear pores [62]. SENP2 also localises to the endoplasmic reticulum [63]. The short isoform of SENP7 (SENP7S) is found in the cytoplasm, whereas the long isoform, SENP7L, is associated with chromatin [64–67]. DeSI1/2 are present in the cytoplasm with DeSI1 also present in the nucleoplasm [43]. SENP3 and SENP5 primarily localise to the nucleolus but have been shown to translocate to the nucleoplasm [68]. SENP6 is found in the nucleoplasm [69]. USPL1 localises to Cajal bodies [70]. Several SENPs can be found within PML nuclear bodies [71].

SUMO proteases also demonstrate substrate specificity through their subcellular localisation, restricting access to subsets of SUMOylated proteins (Figure 3). SUMO protease activity can be modulated by oxidation of catalytic cysteines [72–75], interaction with divalent metal ions [73], and destabilisation following heat shock or hypoxia [48,60].

### SUMO binding domains (SBDs): SIMs, MYMs, zinc fingers and WD40s

**Figure 4:**
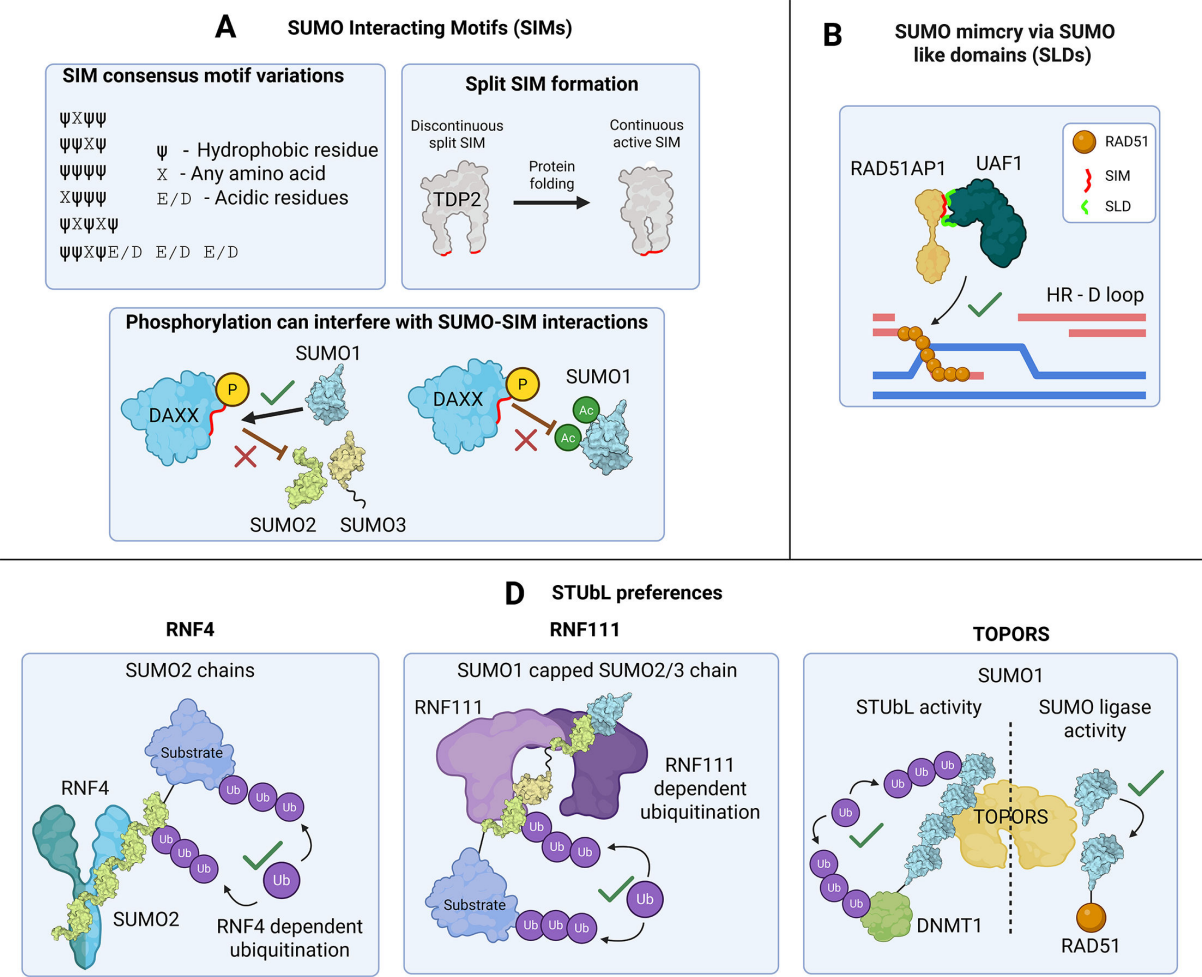
SUMO-binding domains (SBDs). A: The SUMO-interacting motif (SIM). SIMs adopt different variations and orientations but generally include hydrophobic (ψ) and acidic residues (E/D). TDP2 has a split SIM which, through 3D protein folding, orchestrates into a functional SIM. Substrate phosphorylation can interfere with SUMO–SIM interactions as seen with DAXX SUMOylation. SUMO acetylation also has an interference with SUMO–SIM interactions. B: SUMO-like domains (SLD) such as those found in UAF1 can interact with the SIM found in RAD51AP1. This is essential for the formation of the heterotrimeric complex UAF1-RAD51AP1-RAD51 for proper DSB repair via homologous recombination (HR). C: STUbLs recognise SUMO modifications via SIMs and lead to subsequent ubiquitination of the substrate. The STUbLRNF4 interacts with polySUMO2/3 chains. RNF111 interacts with SUMO1 capped SUMO2/3 chains. TOPORS shows SUMO ligase activity in addition to STUbL activity against SUMO1 chains. RAD51AP1, RAD51-associated protein 1; STUbL, SUMO-targeting ubiquitin ligase.

SUMO paralogs and conjugate types can interact with discrete binding domains [7,76–81]. SIMs (Class I SBD) typically consist of three hydrophobic amino acids in various arrangements (Figure 4a) [82–84]. SUMOs interact with SIMs through a hydrophobic groove between the second β-strand and α-helix. SIMs may be flanked by acidic or phosphorylated residues further enhancing electrostatic interactions with positively charged SUMO residues [85]. Despite their simplicity, SIMs can show interaction preference for SUMO paralogs [76]. Phosphorylation of SIM-adjacent residues in DAXX promotes interaction with SUMO1 but not SUMO2/3 [85]. Acetylation of Lys^39^ and Lys^46^ in the SIM-binding groove of SUMO1 reduces interaction with phospho-SIMs from DAXX [17,86]. Therefore, PTMs on both SUMO and substrate can act as molecular discriminators in the SUMOylation system. Multi-mono SUMO binders [87,88] possess multiple SIMs spread throughout their structure, or another yet unidentified SBD [77]. The polySUMO2 interactor, XRCC4, while containing SIMs, interacts through a distinct binding patch on SUMO2 [78,79]. SIMs may also be discontinuous, brought together in their 3D topology – such as the split SIM in TDP2 – making prediction of functional SIMs challenging [89].

SUMO–SIM interactions have roles in generating phase-separated bodies important for partitioning and concentrating proteins [90,91]. SIMs aid substrate recognition and catalysis for E3 trans-SUMOylation [7,92] and cis/intramolecular SUMOylation by CBX4 [93]. Two SIM motifs in ZNF451 play a crucial role in the positioning of SUMOs to favour polySUMOylation [94,95]. Multiple SIM motifs can be found in the unstructured N-termini of SENP6 and SENP7, likely influencing these polySUMO-specific SENPs' ability to recognise their substrates [64,96].

Dual SUMO-ubiquitin interacting modules with tandem SIMs and ubiquitin-interacting motifs (UIMs), such as RAP80, further enhance interaction affinity and recruitment to DSBs enriched with SUMO/Ub modifications. Using sortase-generated Ub-K63 dimers with SUMO2 conjugated at various lysines indicates that K63-Ub_2_-K21-SUMO2 rather than K63-Ub_2_-K11-SUMO2 linkages preferentially interact with the RAP80 SIM-UIM, indicating differential reading of mixed SUMO2 linkages [2,97–99].

MYM zinc finger domains interact with SUMO through the same binding cleft at SIMs, little is known about how these domains interact with SUMO, although proteins containing MYM domains and other zinc fingers are frequently found in SUMO interactomes [7,77–79,100,101]. The Type II SBDs use the E67 patch of SUMO1 for their interaction [102]. SUMO1 cross-linking screening of residues near this patch has identified many other interactors [103]. The Type III interactors use a separate binding mode of SUMO1 and involve ZZ domains found in HERC2 and CBP [104,105].

By comparing pulldown proteomics between SUMO-WT and SUMO-SIM interacting mutants, many proteins were identified that lost SUMO interaction when the SIM-binding groove was disrupted, confirming that a large proportion of the SUMO interactome depends on this surface for interaction [7]. However, some proteins retained interactions with SUMO2/3-SIM mutants, suggesting further uncharacterised SBDs. WD40 domains were prevalent among this class of SIM-independent SUMO interactors, with WD40 domains from SEH1L and SEC13 interacting with a distinct surface of SUMO2 [7].

### SUMO mimics: SLD the SUMO-like domain

Some proteins contain SUMO-like domains (SLDs) that behave as molecular mimics in SUMO signalling (Figure 4b). Two SLDs in USP1-associated factor 1 (UAF1) interact with the SIM in RAD51-associated protein 1 to enhance and stabilise the formation of a trimeric complex with RAD51, which together, in co-operation with BRCA2, generate nucleoprotein filaments required for homologous recombination (HR) repair [106,107]. UAF1s SLD also interact with a SIM in the intra-strand cross-link repair factor FANCI and the PCNA partner ELG1, thus acting to deliver UAF1’s binding partner USP1 to its substrates FANCD2/I-Ub and PCNA-Ub [108]. SLDs in NIP45/NFATC2-interacting protein interact with Ubc9, where they guide specific SUMOylation events critical for DNA catenane resolution and genomic stability [109–111].

### SUMO-targeting ubiquitin ligases (STUbLs): SUMO-Ub generators

The ubiquitin ligase RNF4 has four SIMs, which are required for polySUMO interaction and substrate-induced homodimerisation, which is essential for its Ub-E3 ligase activity [112–115]. RNF111 (Arkadia) is a STUbL identified through motif searches for proteins with tandem SIMs similar to RNF4 [116,117]. RNF111’s SIMs differ from RNF4, preferring SUMO1-capped SUMO2/3 linkages [118]. TOPORS is a dual ubiquitin and SUMO1 E3 ligase that specifically interacts with SUMO1 versus SUMO2/3 [78,119–122]. Recent studies show that TOPORS serves as a SUMO1 STUbL for DNMT1-DNA protein cross-links (DPCs). TOPORS collaborates with RNF4 for clearing SUMOylated DPCs and PML-RARα [123–127]. Additionally, TOPORS promotes SUMO1 modifications of RAD51 and XRCC1, in both cases supporting their respective functions in DNA repair [128,129] (Figure 4c).

### SUMO conjugation waves in DSB-repair signalling

**Figure 5:**
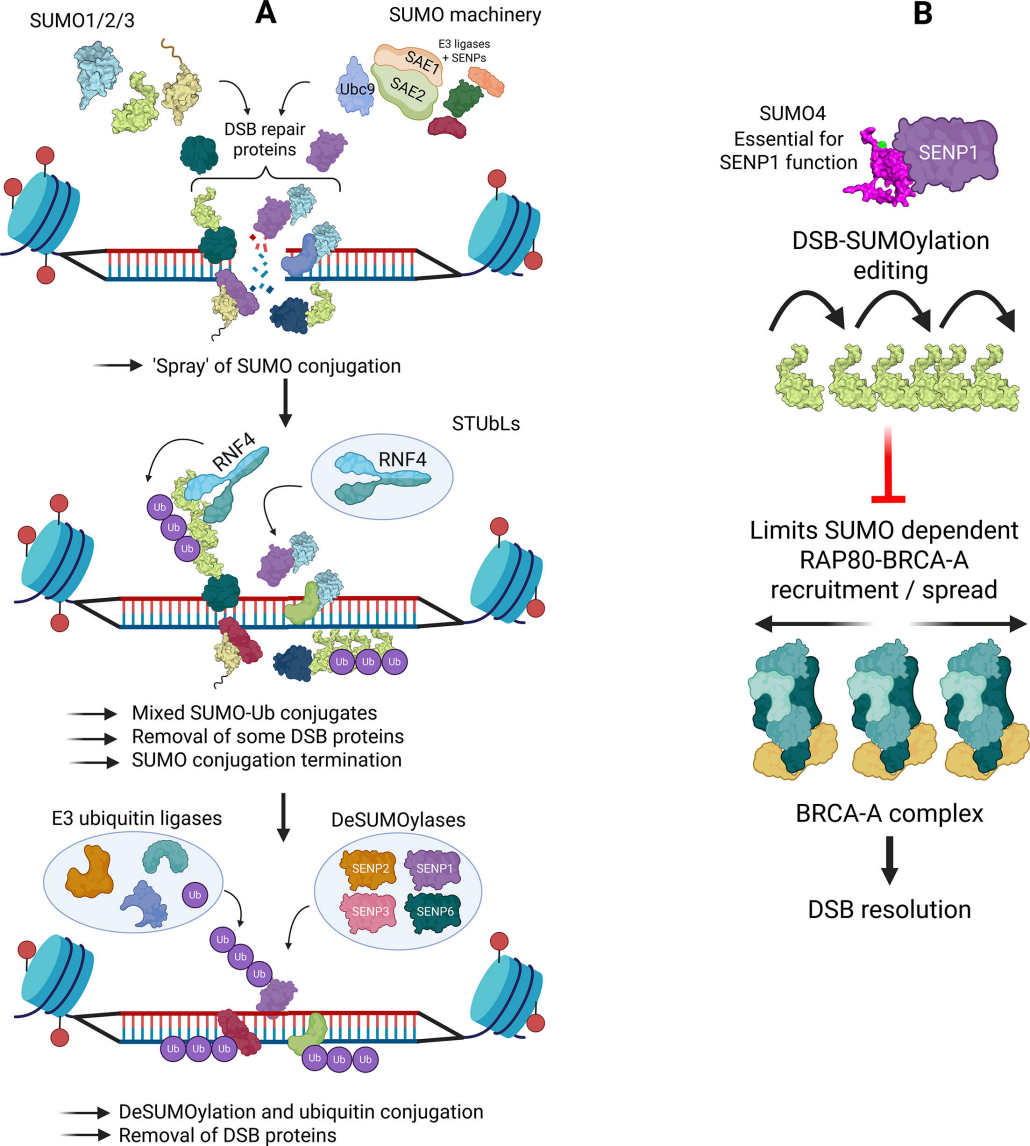
SUMO de/conjugation and SUMO4 in DSB repair. A: The SUMO machinery localisation to DSB sites initiates high levels of SUMO conjugation to various DSB repair proteins (SUMO ‘spray’). This is essential for protein function, localisation, co-operation and clearance. Two types of ubiquitination follow this. The STUbL RNF4 promotes ubiquitination of SUMOylated DSB repair proteins to initiate their clearance and generate mixed SUMO-Ub conjugates. SENP proteases remove or edit SUMO conjugates at DSBs, and ubiquitin E3 ligases generate the ubiquitin conjugates essential for DSB signalling. B: SUMO4 stimulates SENP1 catalytic activity. Optimal SENP1 protease activity is required to prevent excessive SUMOylation at DSBs. It is also essential to limit the recruitment of the SUMO-Ub reader RAP80, which is part of the RAP80/BRCA-A complex. Excessive RAP80 recruitment at DSBs sequesters BRCA1, leading to impaired DNA end resection and DSB repair via HR and increases local concentration of the deubiquitinase BRCC36, which removes K63-Ub linkages read by DSB effectors.

SUMO conjugates have been found to localise directly to DSBs – preceding ubiquitin accumulation (Figure 5a) [69,97,130–138]. For further molecular detail on DSB repair, see [139–141]. The proper recruitment of SUMOylation components is essential for the subsequent recruitment, activity and clearance of the DSB ubiquitination machinery, including RNF168, BRCA1-BARD1 and RAP80-BRCA-A [130,133,136,137,141,142]. SUMO paralogs recruit in waves, with a SUMO1-PIAS4 axis and slightly later SUMO2/3-PIAS1. SUMOylation at DSBs is localised, as the E1-E2-E3 enzymes and some SENPs directly recruit to DSBs [69,131,132]. PIAS1/4 recruits through their DNA-binding SAP domains, CBX4 may recruit through poly-ADP-ribose chains and RNF4 through SIM–SUMO interactions. The recruitment of RNF4 removes several SUMOylated DSB-repair factors and triggers the autoSUMOylation-induced ubiquitination and clearance of SUMO-E3s – shutting down the SUMO conjugation phase of DSB-repair [114]. The concerted action of PIAS4 SUMOylation and RNF4 ubiquitination triggers the VCP/p97-dependent extraction of multiple DSB-localised repair factors [24,137,143,144]. These localised SUMOylation events align with the ‘group SUMOylation model’ established in yeast models of DNA repair signalling, where spatially concentrated SUMO machinery modifies multiple substrates simultaneously [145]. However, at least in mammals, nuances regarding SUMO paralog discrimination, site-specificity, timing, distribution and clearance of SUMOylated DSB-repair factors indicate this is a carefully orchestrated signalling response, not simply a stochastic spray of SUMOs onto co-localised repair factors.

### SUMO deconjugation in DSB repair: amplitude, timing and clearance

The distribution and spreading along chromatin of DSB-repair factors need to be compartmentalised and restricted. DeSUMOylases play essential roles in balancing the burst of SUMOylation signalling that occurs at DSBs. Many DSB-repair factors, such as MDC1, are basally SUMOylated. As one of the earliest recruited DSB factors, localised SUMOylation ultimately promotes RNF4-induced ubiquitination and VCP/p97 extraction of MDC1 from DSBs. SENP2 loss increases MDC1 basal SUMOylation such that upon enrichment at DSBs, MDC1 becomes hyperSUMOylated, resulting in its premature RNF4-VCP-dependent extraction from DSBs. This leads to insufficient recruitment of downstream ubiquitination and repair machinery that rely on MDC1 as a binding platform. Failure to clear MDC1 is also deleterious to DSB repair in non-S-phase cells [130,134,136,137,142]. Multiple DSB-repair proteins, including BRCA1-BARD1, EXO1, BLM, MDC1, RPA70, RIF1, SLX4, RNF168 and 53BP1, are deSUMOylated by SENP6 and SENP1. Disruption of SENP1 or SENP6 results in the mislocalisation of SUMOylated repair factors, causing them to accumulate in phase-separated bodies and thereby preventing their normal DSB relocalisation [69,91,112,130,138]. SENPs can also promote the shutdown of DSB signalling. MRE11A, one of the earliest recruited DSB-repair factors that promotes the initial stages of DNA-end resection, is SUMOylated, which competes with ubiquitination and limits degradation. SENP3 clips these protective SUMOs from MRE11A, thereby triggering ubiquitination/degradation and limiting DSB resection [146]. SENP7, through the deSUMOylation of KAP1, limits SUMO-dependent interaction with the NuRD^CHD3^ complex, promoting chromatin remodelling required for HR [64] (Figure 5a).

### SUMO4, a pseudoSUMO that regulates the amplitude of the DSB-SUMOylation response

SUMO4 disruption results in distinct DSB-repair signalling phenotypes compared with SUMO1–3. SUMO4 down-regulation reduces RNF168 DSB accrual, which consequently impedes accumulation of markers of both major DSB pathways non-homologous end joining (53BP1) and HR (RAD51). SUMO4 mutants that artificially enable SUMO4 conjugation fail to complement DSB-repair defects, indicating that SUMO4 functions independently of conjugation. SUMO4 retains the SIM-binding residues from its SUMO2 ancestor. The SUMO4 SIM-binding mutant does not restore DSB signalling, indicating that SUMO4 has functionally conserved this function [2].

SUMO4-deficient cells have increased SUMO1 and SUMO2/3 conjugates, resulting from impaired SENP1 catalytic activity. *In vitro* SENP1 protease activity is stimulated by pre-incubation with SUMO4. SENP1 also functions in DSB repair [2,64,137,147,148] and SUMO4-SENP1 DSB repair defects are epistatic with each other. Dampening the hyperSUMOylation defect in SUMO4-deficient cells is sufficient to restore DSB signalling, confirming that SUMO4 has a buffering effect on SUMO1–3 conjugates by regulating SENP1 catalytic activity. SENP1 restricts the recruitment of the RAP80 SUMO-Ub reader at DSBs. RAP80 forms part of the BRCA1-A complex (containing BRCA1-BARD1, Abraxas, MERIT40, BRCC45 and BRCC36), which antagonises HR repair by sequestering BRCA1 through Abraxas interaction and by localising the K63-Ub-specific de-ubiquitinase BRCC36. This limits the spreading of ubiquitin-reading DSB-repair factors such as 53BP1. The DSB-repair defects caused by inappropriate recruitment/spread of RAP80 in SUMO4-deficient cells can be restored by disrupting Abraxas-BRCA1 interaction or BRCC36 DUB activity, suggesting these two functions of the BRCA-A complex contribute to the defects observed on SENP1/SUMO4 loss. Therefore, by modulating SENP1 catalytic activity towards the SUMO-conjugates read by RAP80, SUMO4 can tune downstream SUMO-Ub signalling and DSB-repair outcome [2] (Figure 5b).

### SUMOylation of RNF168, recruitment, clearance and phase separation

RNF168 has wide-ranging DSB-repair activities, including histone ubiquitination, chromatin reorganisation and DSB-repair factor recruitment [149]. Canonically, RNF168 is recruited to DSBs through its interaction with histone-ubiquitin conjugates produced by RNF8. ZNF451-dependent SUMOylation of RNF168 can also enable its DSB localisation, promoting downstream histone ubiquitination. This SUMO2ylation of RNF168 protects it from autoubiquitination and degradation [150]. Autoubiquitination of RNF168 and its subsequent degradation tune RNF168 signalling by limiting histone ubiquitination read by RNF168 itself and other DSB-repair factors. Several DUBs deubiquitinate RNF168 to enhance its stability, ultimately modulating the amplitude and distribution of RNF168 signalling on chromatin [151].

At the onset of mitosis, CDK1/2 phosphorylates RNF168 at Thr^208^, creating a phosphothreonine-proline docking site for PIN1. RNF168-Pro^209^ isomerisation then promotes SUMO2ylation at Lys^210^. RNF168^T208A^ or RNF168^K210R^ mutants hyperaccumulate at DSBs, spreading along chromatin. This supraphysiological accumulation of RNF168 on chromatin drives the toxic buildup of the anti-HR repair factor 53BP1, blocking BRCA1-BARD1-dependent DNA end-resection and HR-repair. RNF168 SUMO2ylation enhances its interaction and ubiquitination by RNF4, leading to its extraction from chromatin by the VCP/p97 complex [24]. Earlier findings support this, as RNF168 foci resolution requires RNF4 [133,137], and PIAS4 regulates RNF168 stability [104].

**Figure 6:**
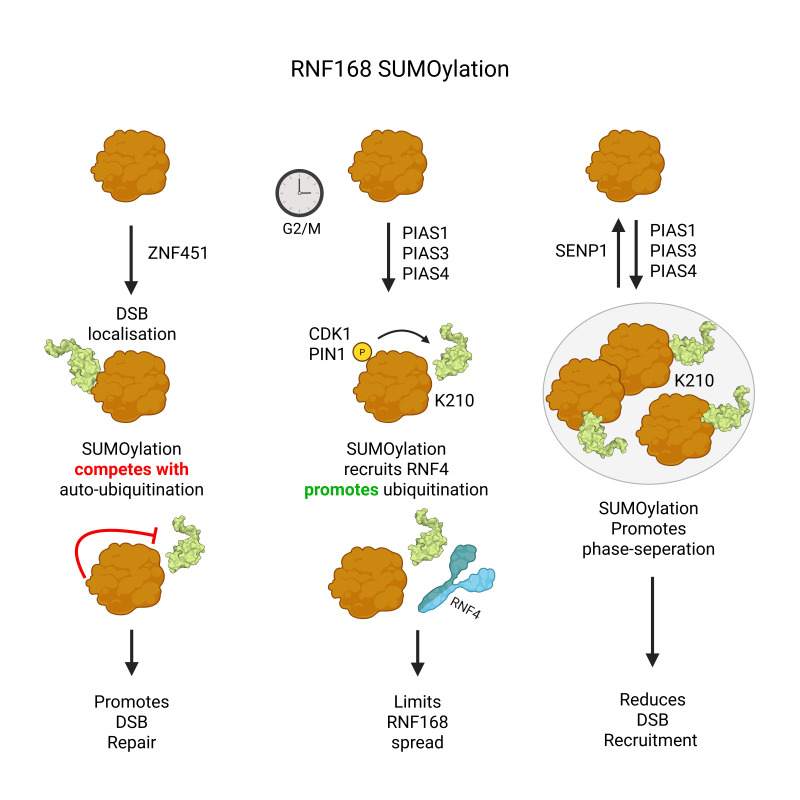
RNF168 SUMOylation and DSB repair. A: RNF168 can be SUMO2ylated by ZNF451, which aids its DSB localisation. SUMOylation competes with autoSUMOylation to prevent RNF168 degradation. B: RNF168 can be phosphorylated at T208 by CDK1/2 during the G2/M transition. This generates a binding site for PIN1 which increases PIAS-dependent SUMO2ylation at K210. RNF168 SUMOylation promotes its RNF4-dependent ubiquitination and VCP-dependent extraction of RNF168 from chromatin. C: RNF168 can be SUMO3ylated. This promotes RN168 phase separation. The isolation of RN168 prevents its recruitment to DSBs and ability to ubiquitinate histones. This process is counteracted by SENP1, which can deSUMOylate RNF168 and allow it to resume normal function.

PIAS1, 3 and 4 each stimulate RNF168^K210^ SUMOylation. Mimicking constitutive SUMO3 modification by genetic fusion drives SUMO3-RNF168 into phase-separated nuclear bodies, which limits RNF168’s ability to recruit to DSBs. This can be reversed by SENP1, which deSUMOylates RNF168 [2,148]. This highlights the importance of maintaining the correct amplitude of RNF168 SUMOylation; too much SUMOylation (by SENP1 inactivation) drives RNF168 into phase-separated bodies and limits access to DSBs, and too little SUMOylation both prevents it from localising and clearing DSBs [24]. In addition to SUMO2ylation discussed here, RNF168 is also SUMO1ylated, although the functional significance (or divergence) between the different paralog modifications is unknown [104]. RNF168 also interacts with K63-Ub-SUMO mixed polymers; how this impacts DSB repair remains to be determined [80] (Figure 6).

### CtIP; multiple SUMOylation sites, multiple functions all converging on HR repair

The endonuclease CtIP is essential for the DNA-end resection steps for HR repair. CBX4 SUMOylates CtIP [152]. Consistent with the previous identification of CtIP SUMOylation across multiple lysines [41,153], the mutation of one SUMOylation site (K896) was insufficient to reduce total CtIP-SUMOylation, but CtIP^K896R^ did display defects in recruitment, DNA resection and RAD51 loading, indicating functional importance of SUMOylation in CtIP function [152].

CtIP also plays a role in protecting the DNA replication fork, a function that overlaps with aspects of its DSB-repair role [154]. One group failed to detect increased CtIP SUMOylation upon IR treatment but did detect PIAS4-dependent CtIP SUMOylation in response to replication stress inducers. This SUMOylation event required CtIP’s phosphorylation by ATR and interaction with PCNA, suggesting that a replication fork-associated fraction of CtIP undergoes SUMOylation. CtIP^K578R^ reduced overall SUMOylation, suggesting it is the dominant site, unlike CtIP^K896R^. However, CtIP^K578R^ mutants maintain MRN and BRCA1 interactions and have normal endonuclease activity *in vitro* yet display defects in HR repair. Mutating K578R significantly affected CtIP replication fork protection activity but not its ability to recruit to DSBs [155].

**Figure 7:**
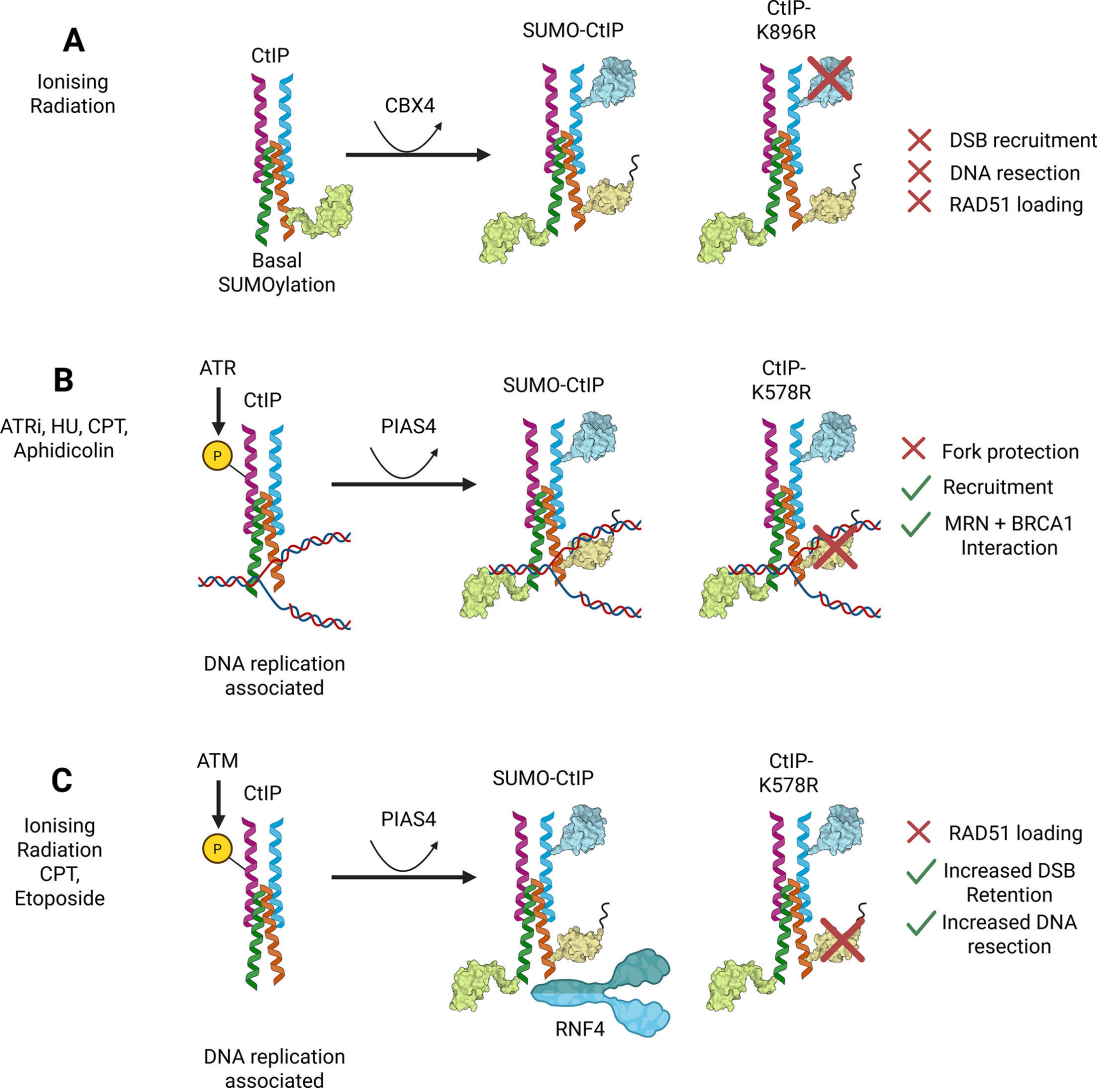
CtIP SUMOylation and DSB repair. A: CtIP is SUMOylated by CBX4 at multiple sites, which are essential for its DSB repair function. CtIP^K896R^ shows reduced DSB localisation and complemented cells show reduced resection and RAD51 loading. B: Replicative-associated CtIP is phosphorylated by ATR. SUMO2ylation of CtIP by PIAS4 can be induced using replication stress inducers: ATR inhibition (ATRi), hydroxyurea (HU), camptothecin (CPT) and aphidicolin. The CtIP^K578R^ mutation results in reduced SUMOylation of CtIP, which causes DNA replication fork protection defects. However, it does not affect CtIP interaction with the MRN complex or BRCA1. C: CtIP can be SUMO2ylated by PIAS4 upon treatment with ionising radiation or etoposide. SUMO2ylation of CtIP promotes interaction with the STUbL RNF4, which mediates CtIP removal via ubiquitin-dependent degradation. Mutation of K578 results in hyperaccumulation of DSBs due to inefficient CtIP clearance and defective DSB repair via HR.

Conversely, another group demonstrated that CtIP SUMOylation is promoted by DSB-inducing agents but not by replication stress. This PIAS4-dependent SUMOylation was responsive to ATM inhibition but not to ATR inhibition. CtIP^K578^ was also identified as a dominant SUMOylation site [156]. SUMOylation and ATM signalling were necessary for CtIP’s interaction with RNF4, which promotes its ubiquitination and degradation. CtIP^K578R^ was observed to hyperaccumulate at DSBs, leading to excessive resection and defective HR repair [156]. A role for SUMOylation in CtIP degradation is further supported by its identification in an RNF4-substrate screen and increased polySUMOylation and degradation in response to SENP6 depletion (Figure 7) [69,114,157].

CtIP, like many SUMO substrates, exists as a pool of molecules modified at different lysines. Single-site mutants such as CtIP^K896R^ may represent a minor subset of the SUMOylated pool, yet show functional impact. The cellular phenotypes of cells expressing mutants of more dominant SUMO acceptors may not match those of other single-site mutants and may functionally diverge depending on the signalling context. To further complicate interpretation, CtIP functions as a tetramer, and it is unknown what combinations of sites and SUMOylation status each monomer contributes to function.

**Summary**

- Despite far fewer components than ubiquitination, small ubiquitin-like modifier (SUMO)ylation is a highly complex modification.
- The SUMO family members have distinct and overlapping functions in cell signalling.
- Many molecular features of the SUMOylation system await further characterisation, such as the specificity of the conjugation and deconjugation components and novel types of SUMO-binding domain.
- SUMOylation can have multiple outcomes on the same DSB repair factor depending on context.

## Abbreviations

ATM: ataxia-telangiectasia mutated
ATR: Ataxia Telangiectasia and Rad3 related
BARD1: BRCA1-Associated Ring Domain protein 1
BLM: Bloom's syndrome
53BP1: p53 binding protein 1
BRCA1: BReast CAncer gene 1
BRCC45: Brain and Reproductive organ Expressed, also known as BRE
CBP: CREB-binding protein
CBX4: chromobox homolog 4
CDK1/2: Cyclin-dependent kinase 1/2
DAXX: Death-associated protein 6
DNMT1: DNA methyltransferase 1
DPC: DNA protein crosslink
DSB: double stranded break
DeSI: deSUMOylating isopeptidase
ELG1: enhanced level of genomic instability 1
EXO1: Exonuclease 1
FANCI: Fanconi anemia, complementation group I
HDAC6: Histone Deacetylase-6
HERC2: HECT And RLD Domain Containing E3 Ubiquitin Protein Ligase 2
HR: homologous recombination
IR: Ionising radiation
KAP1: KRAB-associated protein 1
MDC1: Mediator Of DNA Damage Checkpoint 1
NEDD8: Neural precursor cell expressed developmentally downregulated protein 8
NMR: nuclear magnetic resonance
NSMCE2: NSE2 SUMO Ligase Component Of SMC5/6
PCNA: Proliferating cell nuclear antigen
PELP1: Proline, glutamic acid, and leucine rich protein 1
PIAS1: protein inhibitors of activated STATs 1
PTM: post translational modification
RAD51AP1: RAD51-associated protein 1
RAD51AP1: RAD51 Associated Protein 1
RAP80: receptor-associated protein 80
RIF1: Rap1-interacting factor 1
RNF168: Ring Finger Protein 168
RPA70: Replication protein A 70
SAE2: SUMO-activating enzyme subunit 2
SAP: SAF-A/B, Acinus and PIAS
SBD: SUMO-binding domain
SEH1L: SEH1 Like Nucleoporin
SENP: Sentrin/SUMO Proteases
SIM: SUMO-interacting motif
SLD: SUMO-like domain
SLX4: structure-specific endonuclease subunit 4
SMT3: Suppressor Of Mif Two
STUbL: SUMO-targeting ubiquitin ligase
SUMO: small ubiquitin-like modifier
S. cerevisiae: Saccharomyces cerevisiae
TDP2: Tyrosyl-DNA Phosphodiesterase 2
TOPORS: TOP1 Binding Arginine/Serine Rich Protein
UAF1: USP1-associated factor 1
UIM: ubiquitin-interacting motif
USPL1: ubiquitin-specific protease like 1
VCP: Valosin-containing protein
VSP: Valine, Serine, Proline
XRCC4: X-Ray Repair Cross Complementing 4
ZMIZ2: zinc finger MIZ-type containing 2
ZNF451: Zinc Finger Protein 451
